# Supplementary material for: An adaptive shortest-solution guided decimation approach to sparse high-dimensional linear regression
Source: Sci Rep. 2021 Dec 15;11:24034. doi: 10.1038/s41598-021-03323-7 (PMC8674299; doi:10.1038/s41598-021-03323-7)
Supplement: Supplementary file 1 — Supplementary Information. [file 41598_2021_3323_MOESM1_ESM.pdf]

An adaptive shortest-solution guided decimation approach to sparse high-dimensional linear regression

Xue Yu[1], **Yifan Sun**[1,*], **and Hai-Jun Zhou**[2,3,4,*]

[1]Center for Applied Statistics, School of Statistics, Renmin University of China, Beijing 100872, China
[2]CAS Key Laboratory for Theoretical Physics, Institute of Theoretical Physics, Chinese Academy of Sciences, Beijing 100190, China
[3]School of Physical Sciences, University of Chinese Academy of Sciences, Beijing 100049, China
[4]MinJiang Collaborative Center for Theoretical Physics, MinJiang University, Fuzhou 350108, China
[*]sunyifan@ruc.edu.cn, zhouhj@itp.ac.cn

ABSTRACT

High-dimensional linear regression model is the most popular statistical model for high-dimensional data, but it is quite a challenging task to achieve a sparse set of regression coefficients. In this paper, we propose a simple heuristic algorithm to construct sparse high-dimensional linear regression models, which is adapted from the shortest solution-guided decimation algorithm and is referred to as ASSD. This algorithm constructs the support of regression coefficients under the guidance of the least-squares solution of the recursively decimated linear equations, and it applies an early-stopping criterion and a second-stage thresholding procedure to refine this support. Our extensive numerical results demonstrate that ASSD outperforms LASSO, vector approximate message passing, and two other representative greedy algorithms in solution accuracy and robustness. ASSD is especially suitable for linear regression problems with highly correlated measurement matrices encountered in real-world applications.

A. Properties of estimator $\hat{\boldsymbol{\gamma}}$

According to the Gauss-Markov theorem, we can prove that $\hat{\boldsymbol{\gamma}}$ is the minimum variance linear unbiased estimator of the guidance vector $\boldsymbol{\gamma}$.

Firstly,

$$\hat{\boldsymbol{\gamma}} = \boldsymbol{X}^{+}\boldsymbol{y}.$$

Hence,

$$\mathbb{E}(\hat{\boldsymbol{\gamma}}|\boldsymbol{X}) = \boldsymbol{X}^{+}\mathbb{E}(\boldsymbol{y}|\boldsymbol{X}).$$

Recall that $\boldsymbol{\gamma} = \boldsymbol{X}^{+}\mathbb{E}(\boldsymbol{y}|\boldsymbol{X}) = \boldsymbol{V}_1\boldsymbol{V}_1^{\top}\boldsymbol{\beta}^0$, where the definitions of $\boldsymbol{V}_1$ is provided in article file. Hence $\mathbb{E}(\hat{\boldsymbol{\gamma}}|\boldsymbol{X}) = \boldsymbol{\gamma}$. Therefore, $\hat{\boldsymbol{\gamma}}$ is an unbiased estimator of $\boldsymbol{\beta}^0$. Next, to show $\hat{\boldsymbol{\gamma}}$ is BLUE, we need to compare $\hat{\boldsymbol{\gamma}}$ with all other linear unbiased estimator of $\boldsymbol{\gamma}$. Any estimator of this class can be written as

$$\boldsymbol{\gamma}_0 = \boldsymbol{C}\boldsymbol{y},$$

where $\boldsymbol{C}$ is a $p \times n$ matrix that does not depend on either $\boldsymbol{y}$ or $\boldsymbol{\gamma}$. Since we assume that $\boldsymbol{\gamma}_0$ is unbiased, then it follows that

$$\mathbb{E}(\boldsymbol{\gamma}_0|\boldsymbol{X}) = \mathbb{E}(\boldsymbol{C}\boldsymbol{y}|\boldsymbol{X}) = \boldsymbol{C}\mathbb{E}(\boldsymbol{y}|\boldsymbol{X}) = \boldsymbol{C}\boldsymbol{X}\boldsymbol{\beta}^0 = \boldsymbol{\gamma} = \boldsymbol{V}_1\boldsymbol{V}_1^{\top}\boldsymbol{\beta}^0,$$

which implies that $\boldsymbol{C}\boldsymbol{X} = \boldsymbol{V}_1\boldsymbol{V}_1^{\top}$.

For $\hat{\boldsymbol{\gamma}}$ to be BLUE, we need to establish that $\mathrm{Var}(\boldsymbol{\gamma}_0|\boldsymbol{X}) - \mathrm{Var}(\hat{\boldsymbol{\gamma}}|\boldsymbol{X})$ is positive semidefinite. Note that

$$\begin{aligned}\mathrm{Var}(\boldsymbol{\gamma}_0|\boldsymbol{X}) &= \mathbb{E}[(\boldsymbol{C}\boldsymbol{y} - \hat{\boldsymbol{\gamma}} + \hat{\boldsymbol{\gamma}} - \boldsymbol{\gamma})(\boldsymbol{C}\boldsymbol{y} - \hat{\boldsymbol{\gamma}} + \hat{\boldsymbol{\gamma}} - \boldsymbol{\gamma})^{\top}|\boldsymbol{X}] \\ &= \mathbb{E}[(\boldsymbol{C}\boldsymbol{y} - \hat{\boldsymbol{\gamma}})(\boldsymbol{C}\boldsymbol{y} - \hat{\boldsymbol{\gamma}})^{\top}|\boldsymbol{X}] + \mathrm{Var}(\hat{\boldsymbol{\gamma}}|\boldsymbol{X}) + 2\mathbb{E}[(\boldsymbol{C}\boldsymbol{y} - \hat{\boldsymbol{\gamma}})(\hat{\boldsymbol{\gamma}} - \boldsymbol{\gamma})^{\top}|\boldsymbol{X}].\end{aligned}$$

Because $\mathbb{E}(\boldsymbol{C}\boldsymbol{y} - \hat{\boldsymbol{\gamma}}|\boldsymbol{X}) = 0$, we have

$$
\begin{aligned}
\mathbb{E}[(\boldsymbol{C}\boldsymbol{y}-\hat{\boldsymbol{\gamma}})(\hat{\boldsymbol{\gamma}}-\boldsymbol{\gamma})^\top|\boldsymbol{X}] &= \mathrm{cov}(\boldsymbol{C}\boldsymbol{y}-\hat{\boldsymbol{\gamma}},\hat{\boldsymbol{\gamma}}|X) \\
&= \mathrm{cov}(\boldsymbol{C}\boldsymbol{y}-\boldsymbol{X}^+\boldsymbol{y},\boldsymbol{X}^+\boldsymbol{y}|\boldsymbol{X}) \\
&= \mathrm{cov}([\boldsymbol{C}-\boldsymbol{X}^+]\boldsymbol{y},\boldsymbol{X}^+\boldsymbol{y}|\boldsymbol{X}) \\
&= (\boldsymbol{C}-\boldsymbol{X}^+)\mathrm{Var}(\boldsymbol{y}|\boldsymbol{X})(\boldsymbol{X}^+)^\top \\
&= \sigma^2(\boldsymbol{C}-\boldsymbol{X}^+)\boldsymbol{X}(\boldsymbol{X}^\top\boldsymbol{X})^+ \\
&= \sigma^2(\boldsymbol{C}\boldsymbol{X}-\boldsymbol{X}^+\boldsymbol{X})(\boldsymbol{X}^\top\boldsymbol{X})^+ \\
&= 0.
\end{aligned}
\tag{1}
$$

where the last equation holds because of $\boldsymbol{X}^+\boldsymbol{X}=\boldsymbol{V}_1\boldsymbol{V}_1^\top$ and $\boldsymbol{C}\boldsymbol{X}=\boldsymbol{V}_1\boldsymbol{V}_1^\top$. Combined with equation (1), we obtain that $\mathrm{Var}(\boldsymbol{\gamma_0}|\boldsymbol{X}) \geq \mathrm{Var}(\hat{\boldsymbol{\gamma}}|\boldsymbol{X})$, and the equation holds if and only if $\boldsymbol{\gamma_0}=\hat{\boldsymbol{\gamma}}$.

B. The calculation process of estimator $\hat{\boldsymbol{\gamma}}$

In fact, the estimated guidance vector $\hat{\boldsymbol{\gamma}}$ not only is a least squares solution of the original linear model, but the one with minimum length (minimum Euclidean norm). As such, $\hat{\boldsymbol{\gamma}}$ can be obtained through convex minimization with the following objective function

$$
\frac{1}{2}\hat{\boldsymbol{\gamma}}^2+\boldsymbol{\lambda}^\top(\boldsymbol{y}-\boldsymbol{X}\hat{\boldsymbol{\gamma}}),
\tag{2}
$$

where $\boldsymbol{\lambda}^\top=(\lambda_1,\lambda_2,...,\lambda_n)^\top$ is a column vector of Lagrange multipliers $\lambda_i(i=1,2,...,n)$.

This problem can be solved by using dual ascent method:

$$
\begin{aligned}
\hat{\boldsymbol{\gamma}}^{(t)} &= \boldsymbol{X}^\top\boldsymbol{\lambda}^{(t)}, \\
\boldsymbol{\lambda}^{(t+1)} &= \boldsymbol{\lambda}^{(t)}+\varepsilon^{(t)}(\boldsymbol{y}-\boldsymbol{X}\hat{\boldsymbol{\gamma}}^{(t)}),
\end{aligned}
\tag{3}
$$

At each iteration step $t(=0,1,\ldots)$, the step-size $\varepsilon^{(t)}$ is set to an optimal value to minimize the convergence time. Specifically, define $\boldsymbol{e}^{(t)}=\boldsymbol{y}-\boldsymbol{X}\hat{\boldsymbol{\gamma}}^{(t)}$. Then after the $(t+1)$-th iteration step, we have

$$
\begin{aligned}
\boldsymbol{\lambda}^{(t+1)} &= \boldsymbol{\lambda}^{(t)}+\varepsilon^{(t)}\boldsymbol{e}^{(t)}, \\
\boldsymbol{e}^{(t+1)} &= \boldsymbol{y}-\boldsymbol{X}\hat{\boldsymbol{\gamma}}^{(t+1)} \\
&= \boldsymbol{y}-\boldsymbol{X}\boldsymbol{X}^\top\boldsymbol{\lambda}^{(t+1)} \\
&= \boldsymbol{y}-\boldsymbol{X}\boldsymbol{X}^\top(\boldsymbol{\lambda}^{(t)}+\varepsilon^{(t)}\boldsymbol{e}^{(t)}) \\
&= \boldsymbol{e}^{(t)}-\varepsilon^{(t)}\boldsymbol{X}\boldsymbol{X}^\top\boldsymbol{e}^{(t)}.
\end{aligned}
\tag{4}
$$

Let $\boldsymbol{\eta}^{(t)}=\boldsymbol{X}\boldsymbol{X}^\top\boldsymbol{e}^{(t)}$, and then $\boldsymbol{e}^{(t+1)}=\boldsymbol{e}^{(t)}-\varepsilon^{(t)}\boldsymbol{\eta}^{(t)}$. The Euclidean norm (i.e., l_2 norm) of residual $\boldsymbol{e}^{(t+1)}$ is minimized by setting

$$
\varepsilon^{(t)}=\frac{\langle\boldsymbol{e}^{(t)},\boldsymbol{\eta}^{(t)}\rangle}{\langle\boldsymbol{\eta}^{(t)},\boldsymbol{\eta}^{(t)}\rangle}.
\tag{5}
$$

Therefore, the optimal $\varepsilon^{(t)}$ in equation (5) is used in the dual descent process.

C. Additional numerical results

Table S1. Simulation results on a real-world measurement matrix with $p = 2000$, $n = 300$, $\sigma^2 = 1$ and $s_0 = 40$. The true nonzero coefficients are sampled from the uniform distribution $\mathscr{U}[0.5, 1]$ and $\mathscr{U}[-1, -0.5]$ with equal probability.

Methods	TP	FP	RE	Time
LASSO	38(2.00)	119(18.99)	5.09E-01(8.66E-02)	20.00(2.31)
ALASSO	38(2.02)	105(17.69)	4.59E-01(7.91E-02)	29.29(4.01)
VAMP	**39(3.04)**	358(305.38)	3.42E-00(2.87E+01)	**0.09(0.06)**
SIS+LASSO	8(2.52)	20(3.76)	9.41E-01(3.48E-02)	1.72(0.83)
ASDAR	35(6.66)	18(14.30)	4.01E-01(2.51E-01)	0.20(0.10)
ASSD	35(4.63)	**9(7.74)**	**3.92E-01(1.97E-01)**	16.00(1.34)

Table S2. Simulation results on a real-world measurement matrix with $p = 2000$, $n = 300$, $\sigma^2 = 1$ and $s_0 = 40$.The true nonzero coefficients are sampled from uniform distribution $\mathscr{U}[0.2, 1]$.

Methods	TP	FP	RE	Time
LASSO	35(2.56)	104(17.79)	4.98E-01(6.37E-02)	18.85(1.89)
ALASSO	34(2.63)	92(16.18)	4.64E-01(5.49E-02)	26.21(3.07)
VAMP	**39(1.17)**	156(208.87)	8.80E-00(8.39E+01)	**0.11(0.06)**
SIS+LASSO	7(2.21)	19(4.35)	9.12E-01(4.84E-02)	1.48(0.66)
ASDAR	31(4.35)	13(8.27)	**4.21E-01(1.48E-01)**	0.16(0.06)
ASSD	29(3.95)	**12(7.45)**	4.85E-01(1.43E-01)	15.64(1.37)

Table S3. Simulation results on a real-world measurement matrix with $p = 2000$, $n = 300$, $\sigma^2 = 1$. The $s_0 = 40$ coefficients are sampled from uniform distribution $\mathscr{U}[0.5, 1]$, and other coefficients are set to be 0.001.

Methods	TP	FP	RE	Time
LASSO	39(1.21)	124(16.32)	4.64E-01(6.77E-02)	24.98(3.41)
ALASSO	39(1.23)	110(15.17)	4.29E-01(6.55E-02)	36.50(5.19)
VAMP	**40(0.39)**	71(340.78)	2.19E+01(1.93E+02)	**0.14(0.07)**
SIS+LASSO	7(2.19)	19(3.77)	9.44E-01(3.43E-02)	1.57(0.77)
ASDAR	36(4.61)	16(13.77)	3.71E-01(2.22E-01)	0.26(0.10)
ASSD	36(4.20)	**8(7.85)**	**3.45E-01(1.85E-01)**	17.05(2.49)

Table S4. Simulation results on a real-world measurement matrix with $p = 2000$, $n = 300$, $\sigma^2 = 1.5$ and $s_0 = 40$.

Methods	TP	FP	RE	Time
LASSO	37(2.61)	110(17.78)	5.65E-01(9.09E-02)	34.64(3.53)
ALASSO	37(2.64)	97(15.68)	**5.23E-01(9.22E-02)**	50.26(6.60)
VAMP	**40(0.49)**	79(293.71)	7.26E-00(6.95E+01)	**0.16(0.07)**
SIS+LASSO	7(2.33)	20(4.02)	9.48E-01(3.76E-02)	2.17(0.75)
ASDAR	32(6.67)	21(13.62)	5.64E-01(2.54E-01)	0.31(0.11)
ASSD	30(5.49)	**19(7.44)**	6.22E-01(2.00E-01)	33.24(3.01)

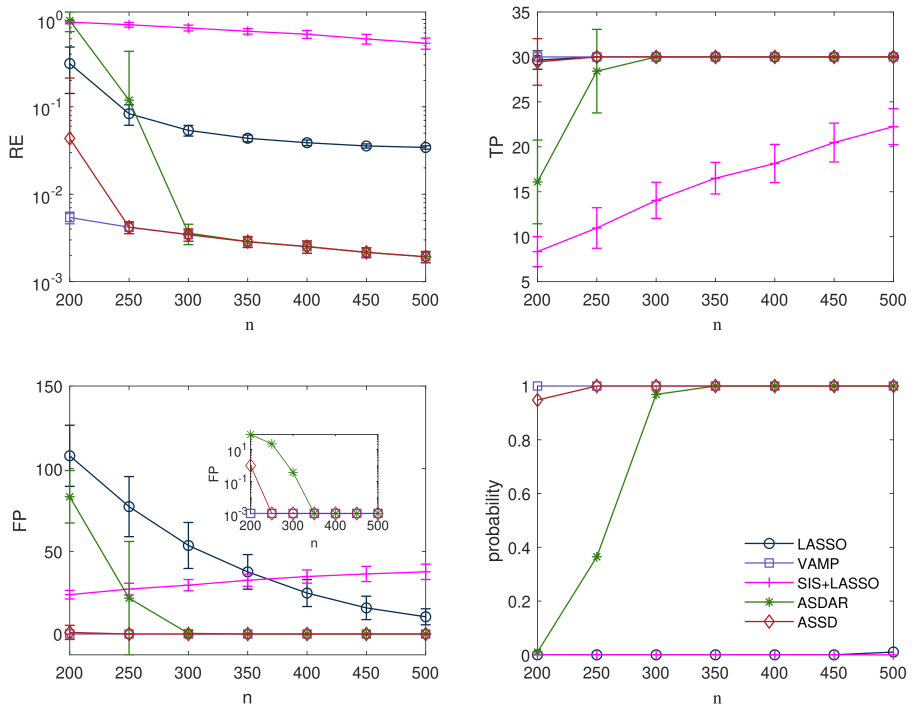

Supplementary Figure S1. Simulation results on the structured measurement matrix: influence of the sample size n ($p = 2000$, $s_0 = 30$, $\sigma^2 = 0.5$). Relative errors (top left); true positives (top right); false positives (bottom left) with the inset being a semi-logarithmic plot, probability of exact identification of nonzero coefficients (bottom right).

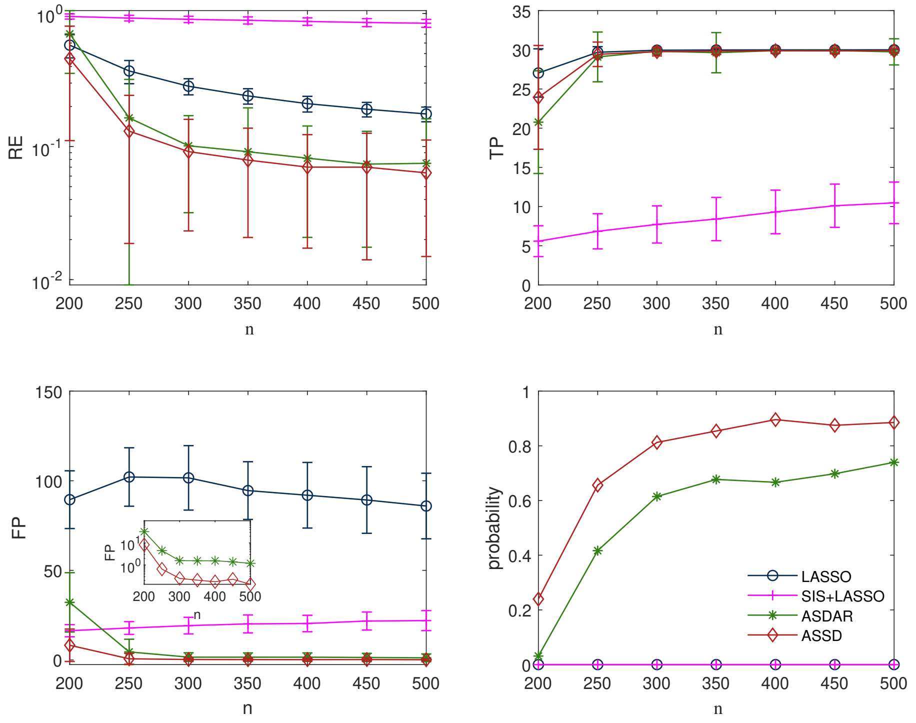

Supplementary Figure S2. Simulation results on the real-world measurement matrix: influence of the sample size n ($p = 2000$, $s_0 = 30$, $\sigma^2 = 0.5$). Relative errors (top left); true positives (top right); false positives (bottom left) with the inset being a semi-logarithmic plot; probability of exact identification of nonzero coefficients (bottom right).

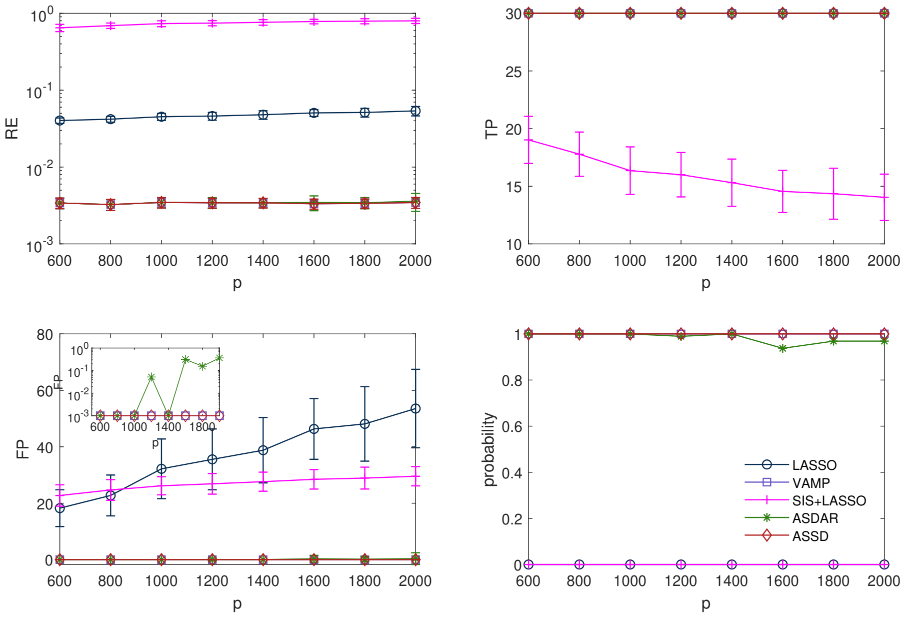

Supplementary Figure S3. Simulation results on the structured measurement matrix: influence of the covariates number p ($n = 300$, $s_0 = 30$, $\sigma^2 = 0.5$). Relative errors (top left); true positives (top right); false positives (bottom left) with the inset being a semi-logarithmic plot; probability of exact identification of nonzero coefficients (bottom right).

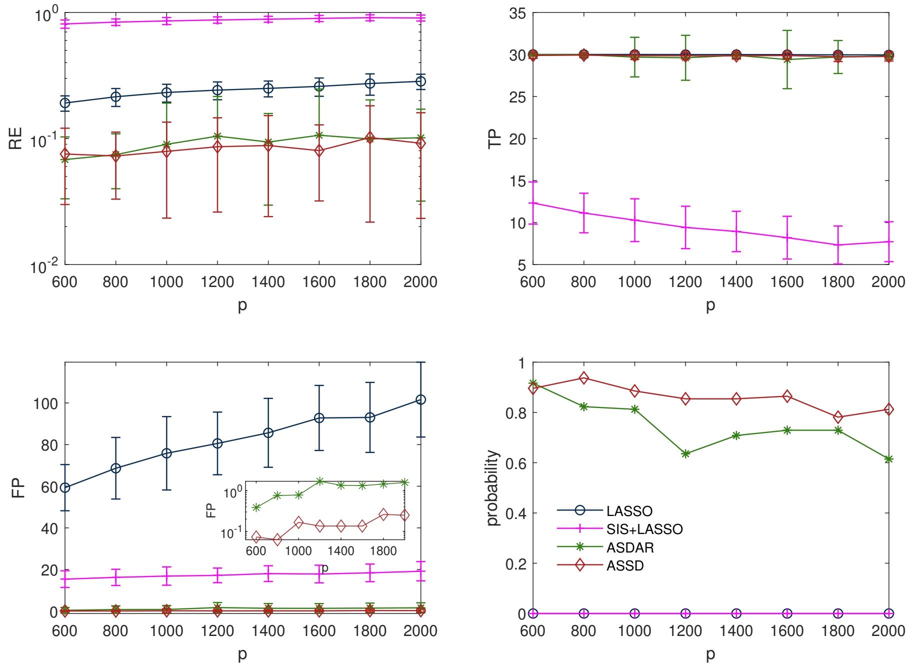

Supplementary Figure S4. Simulation results on the real-world measurement matrix: influence of the covariates number p ($n = 300$, $s_0 = 30$, $\sigma^2 = 0.5$). Relative errors (top left); true positives (top right); false positives (bottom left) with the inset being a semi-logarithmic plot; probability of exact identification of nonzero coefficients (bottom right).

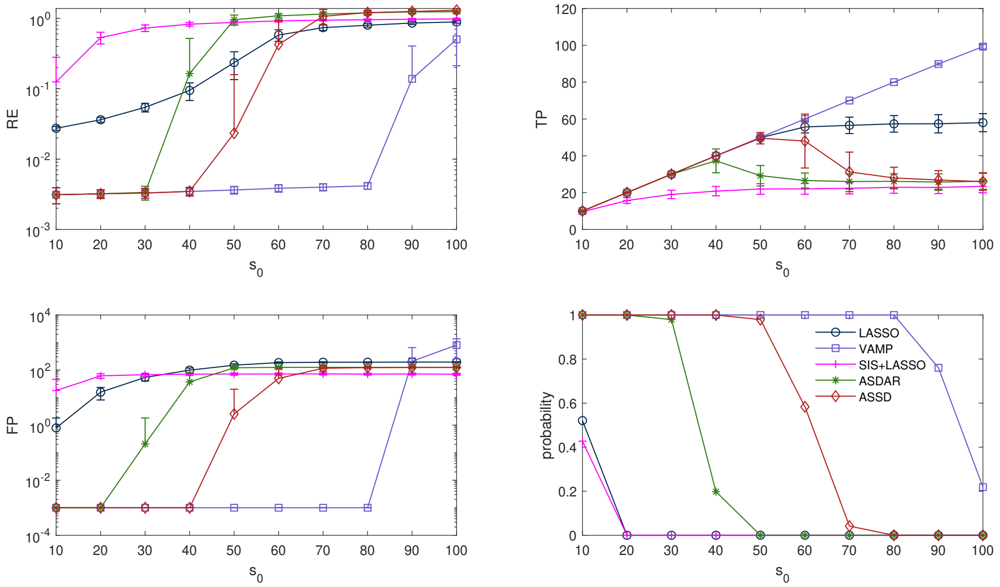

Supplementary Figure S5. Simulation results on the structured measurement matrix: influence of the sparsity level s_0 ($n = 300$, $p = 2000$, $\sigma^2 = 0.5$). Relative errors (top left); true positives (top right); false positives (bottom left) with the inset being a semi-logarithmic plot; probability of exact identification of nonzero coefficients (bottom right).

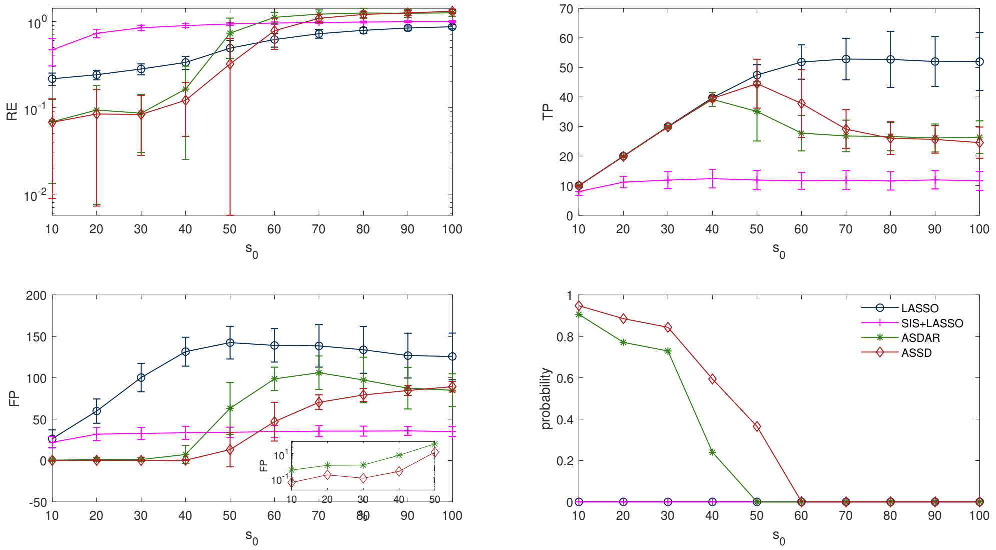

Supplementary Figure S6. Simulation results on the real-world measurement matrix: the influence of the sparsity level s_0 ($n = 300$, $p = 2000$, $\sigma^2 = 0.5$). Relative errors (top left); true positives (top right); false positives (bottom left) with the inset being a semi-logarithmic plot; probability of exact identification of nonzero coefficients (bottom right).
